# Fetal Multicystic Dysplastic Kidney and Its Effect on Post-natal Quality of Life: A Literature Review

**DOI:** 10.7759/cureus.110488

**Published:** 2026-06-08

**Authors:** Kuragamage Dona Prabuddhi Thiloka Kuragama, Mikhail Johnson, Mustafa Abrar Zaman, Jyothsnaa Sathish, Aseel Aljeshi, Sweety Akter, John Shields

**Affiliations:** 1 Surgery, St. George's University School of Medicine, St. George's, GRD; 2 Anesthesiology, St. George's University School of Medicine, St. George's, GRD; 3 Cardiology, St. George's University School of Medicine, St. George's, GRD; 4 Emergency Medicine, St. George's University School of Medicine, St. George's, GRD; 5 School of Medicine, St. George's University School of Medicine, St. George's, GRD; 6 Biotechnology, BRAC University, Dhaka, BGD; 7 Urology, State University of New York (SUNY) Downstate Health Sciences University, New York, USA

**Keywords:** congenital anomalies of the kidney and urinary tract (cakut), fetal renal failure (frf), multicystic dysplastic kidney (mcdk), pediatric nephrology, prenatal diagnosis

## Abstract

Multicystic dysplastic kidney (MCDK) is a congenital renal malformation defined by a nonfunctional kidney consisting of numerous cysts and dysplastic parenchyma. MCDK most commonly affects one kidney that follows a benign course with spontaneous involution and compensatory hypertrophy of the contralateral kidney. Recent advances in prenatal ultrasonography and genetic testing such as chromosomal microarray analysis have improved early detection and risk stratification, allowing for individualized management approaches. Current guidelines favor conservative management for simple unilateral cases, prioritizing periodic blood pressure monitoring, renal function assessment, and surveillance for rare complications; these include hypertension, urinary tract infections, and malignancy. Complex or bilateral presentations have an increased risk of chronic kidney disease and perinatal morbidity, sometimes requiring multidisciplinary management, surgical intervention, or experimental fetal therapies. Beyond clinical outcomes, MCDK management has significant psychosocial, ethical, and economic implications for affected families. This review discusses current evidence on the pathophysiology, prenatal diagnosis, management approaches, risk stratification, and long-term clinical and psychosocial outcomes associated with MCDK.

## Introduction and background

Multicystic dysplastic kidney (MCDK) is a congenital renal disorder characterized by a nonfunctional, lobulated kidney with noncommunicating cysts and abnormal parenchymal tissue. MCDK affects 1 in 1,000-4,000 live births and is one of the most common causes of abdominal masses in newborns. Unilateral and isolated MCDK cases have favorable outcomes, demonstrating a tendency for spontaneous involution, where the cystic kidney may decrease in size or resolve entirely over time [1]. Alternatively, bilateral involvement is seen in 1 in 10,000 MCDK cases, with a male preponderance. Bilateral MCDK may cause fetal renal failure (FRF) and carries a worse prognosis [2-4]. During normal nephrogenesis, there are three important structures: pronephros, mesonephros, and metanephros. Pronephros does not have functional relevance, while mesonephros does not have enduring renal function. The most caudal part of the mesonephros, i.e., the ureteric bud, invades the metanephric mesenchyma and, through reciprocal induction, the ureteric bud becomes the urinary collecting system, while the metanephric mesenchyma undergoes the mesenchymal to epithelial transition, ultimately becoming the rest of the functional nephron [5,6]. MCDK is thought to occur due to disruptions during renal embryogenesis, resulting in abnormal development and interactions between the ureteric bud and renal mesenchyme [7].

Detection of MCDK is incidental and occurs during routine prenatal ultrasonography, facilitating effective and timely decision-making and parental counselling [8,9]. Current management guidelines shifted away from routine nephrectomy towards a conservative approach. In the past, nephrectomies were common due to unjustified concerns of pain, hypertension, infection, and malignant transformation [10]. Now, surgical intervention is indicated solely for complex cases of MCDK. Bilateral MCDK shows high rates of morbidity and mortality due to FRF, and hence, emerging experimental interventions such as serial amnioinfusions followed by neonatal dialysis and renal transplantation have been explored in recent studies [4,11]. These approaches remain investigational and raise important ethical, clinical, and psychosocial questions [4,11]. This literature review will discuss these considerations in regard to long-term monitoring requirements, early detection, risk stratification, and management of MCDK, in addition to the psychosocial and financial implications of MCDK on their patients and families.

## Review

Methods 

Our review is a narrative literature review without an intention to be perceived as a systematic review or a meta-analysis. We focused on the psychosocial aspects of a novel intervention for the treatment of bilateral MCDK that causes FRF, using the study by Miller et al. as the landmark study [11]. Using backward citation, we found Bienstock et al., which was the first study published in 2014 to report a successful use of in utero interventions for FRF [12]. Our team used the PubMed search engine to identify pertinent studies that used amnioinfusion therapies and transplantation for FRF in the following publication date ranges: 2014-2025. Our chief limitation is the small sample size (only eight studies, including case reports and case series); however, our study is the first, to our knowledge, delving into the psychosocial outcomes in patients who received these interventions. Perhaps, future researchers can use the modest data gathered in our review to present a more comprehensive overview of these outcomes.

Etiology and pathophysiology

MCDK is a multifactorial congenital disorder resulting from abnormal genetic and developmental interactions during nephrogenesis, which is confirmed by cytogenomics in 30% of cases [13]. Chromosomal microarray (CMA) reveals pathogenic copy number variations in 13.5-16.7% of cases, including recurrent deletions at 17q12, linked to renal cysts and diabetes syndrome [14]. Other deletions or duplications at loci 22q11.21 and genes BBS9 and BMPER located at 7p14.3 have also been implicated in MCDK development [14-16]. If CMA results are negative, next-generation sequencing or whole genome sequencing may identify monogenic causes, i.e., mutations in PAX2, EYA1, and SIX1, which account for 13-19% of cases [15]. These genes provide the basis for the ureteric bud theory, the principal developmental explanation for MCDK. It proposes that MCDK arises from abnormal branching of the ureteric bud and its disrupted interaction with the surrounding metanephric mesenchyme. Genes previously discussed encode transcription factors that facilitate these interactions [17].

Current guidelines recommend genetic testing in cases of bilateral MCDK and in unilateral cases associated with extrarenal anomalies [18]. Mutations in PAX2 are associated with renal coloboma syndrome, warranting ophthalmologic evaluation postnatally, whereas variants in EYA1 or SIX1 are associated with branchio-oto renal syndrome, necessitating assessment for branchial arch anomalies and audiologic evaluation [19]. Deletions involving HNF1β require surveillance for maturity-onset diabetes of the young type 5 (MODY5) and autism spectrum disorder [18]. Identification of a genetic etiology increases prognostic accuracy and guides risk stratification for progressive renal dysfunction [20]. 

According to Gimpel et al., identification of a molecular level etiology facilitates genetic counselling and supports shared decision making regarding prenatal and postnatal management, as well as risk of recurrence in future pregnancies, estimated at 10-25%. Confirmation of a genetic contribution reduces the need for invasive diagnostic procedures such as renal biopsies and influences prenatal counseling and pregnancy management when significant prognostic implications are identified antenatally [18].

Prenatal diagnosis of MCDK

Early identification and management of MCDK rely primarily on prenatal diagnosis. The main diagnostic method is renal and bladder ultrasound (RBUS) as it can detect renal lesions during routine prenatal imaging around 20 weeks of gestation [7,9]. Postnatal imaging is required to confirm the diagnosis and assess contralateral kidney function. RBUS is sufficient at diagnosing MCDK with a sensitivity and specificity of 99% and 99.9%, respectively [21]. Renal studies such as dimercaptosuccinic acid or technetium 99m diethylenetriaminepentacetate scintigraphy are not recommended, especially when the contralateral kidney is normal [22]. Definitions are important when discussing MCDK. A unilateral MCDK without any other genitourinary abnormalities is a simple MCDK, whereas a unilateral MCDK with genitourinary abnormalities is a complex MCDK [1]. Bilateral MCDK falls under the FRF syndrome and is considered lethal [9]. However, recent advancements in serial amnioinfusions and neonatal dialysis have demonstrated isolated success and improved prognostic outcomes [11]. Karyotyping is not usually necessary for isolated unilateral MCDK; CMA may be taken into consideration in more complex presentations of the disease due to possible associations with aneuploidy or systemic involvement [8]. Early diagnosis is critical for prognosis, clinical decision-making, and offering families the necessary time for care planning and counselling.

Current guidelines for the management of MCDK

Simple MCDK: Conservative Management

Nephrectomy was common for MCDK due to concerns for rare complications such as malignancy and hypertension (HTN). Currently, conservative management is favored as MCDK demonstrates a tendency to involute with age. The rate of involution is 10% by one year, 35% by two years, 38-47% by five years, and 60% at 10-15 years of age [10]. Some authors rely on the cranio-caudal size of the lesion, emphasizing that lesions larger than 50 mm tend not to involute; the greatest rates of involution occur in the first 30 months of life [1,10]. In their cohort of 72 cases, Faruque et al. found that in the six-month period between the initial and subsequent postnatal RBUS, a maximal cranio-caudal reduction of the MCDK of greater than 20% was predictive of complete involution [23]. 

Reduced function of the affected kidney in simple MCDK necessitates the monitoring of hypertrophy of the contralateral kidney [22]. It is recommended to solely use ultrasound surveillance and avoid renal scintigraphy studies [22]. Ninety-five percent of patients who develop compensatory hypertrophy do so by the age of three years; however, 10% of MCDK cases do not compensate by the age of ten [22]. Absence of hypertrophy in the contralateral kidney is explained through a “vascular steal” mechanism, whereby blood is shunted toward the affected kidney, reducing the perfusion of the healthy kidney [23]. 

Complications that may develop due to simple MCDK include HTN, urinary tract infections (UTIs), and malignancies such as Wilms tumors (WTs) [1,23,24]. HTN develops in 3.2% of cases [23]. UTIs are seen in 8.8.% of children with normal contralateral RBUS and in 13.2% of children with an abnormal contralateral ultrasound. On the contrary, UTIs are reported in 7.8% of healthy children [24]. Malignant transformation is seen in 0.04-0.07% of cases [1,10]. Therefore, simple MCDK follows a benign course comparable to that of the general pediatric population, allowing a conservative management approach when resolution is confirmed.

Monitoring Protocols

When complete resolution of MCDK does not occur, both the affected kidney and the healthy kidney require monitoring [23]. Local practices differ over monitoring intervals. Alamir et al. highlight the importance of performing a complete check-up consisting of blood pressure monitoring, abdominal examination, and review of renal laboratory studies such as eGFR, creatinine, and urinalysis [25]. RBUS monitoring for the involution and hypertrophy of the contralateral kidney is highly advised, with involution signifying resolution of the lesion [1]. Faruque and colleagues recommend performing surveillance on an annual basis, while Briggs and colleagues recommend ultrasound at specific intervals, i.e., birth, six months, and two, five, 10, and 15 years [1,23]. Follow-up compliance is critical due to the risk of developing chronic kidney disease (CKD) in children before age 10 [1]. Caregiver education is a critical component of improving follow-up adherence. CKD is a risk factor as well as a consequence of HTN [26], highlighting that the absence of HTN does not imply the absence of reduced kidney function. Lack of involution, development of complications, or lack of contralateral kidney compensatory hypertrophy may warrant more extensive intervention, such as nephrectomy.

Surgical Considerations: Complex MCDK

Complex MCDK is the co-occurrence of MCDK with additional genitourinary abnormalities, seen in 30-50% of cases [9]. The most common abnormality is vesicoureteral reflux (VUR) in the contralateral kidney, seen in 16-21% of cases [27]. Other abnormalities include duplex systems, ureteroceles, agenesis, ectopia, posterior urethral valves, and pelvi-ureteral junction obstruction [9,23]. Across 37 studies evaluating 303 patients, Erlich et al. found that 68% of VURs spontaneously downgraded to Grade I VUR, and ureteral re-implantation was performed in 14% of cases; CKD was noted in two cases [28]. This suggests that over half of all patients with VUR, including dilating (Grade III-V) VUR, resolve spontaneously, while grave sequelae such as CKD are rare. This underscores the need for familial shared decision-making regarding the potential incorporation of invasive investigations, such as voiding cystourethrograms (VCUGs) for surveillance of VUR and possible surgical correction. Kopac and Alamir advise against routine screening using VCUG due to low clinical significance, recommending it only in cases of UTIs or ultrasonographic findings suggesting VUR dilatation [25,27]. Faruque et al. state that if the concurrent anomalies need to be surgically corrected along with an MCDK nephrectomy, both procedures can be performed concurrently [23].

**Figure 1:**
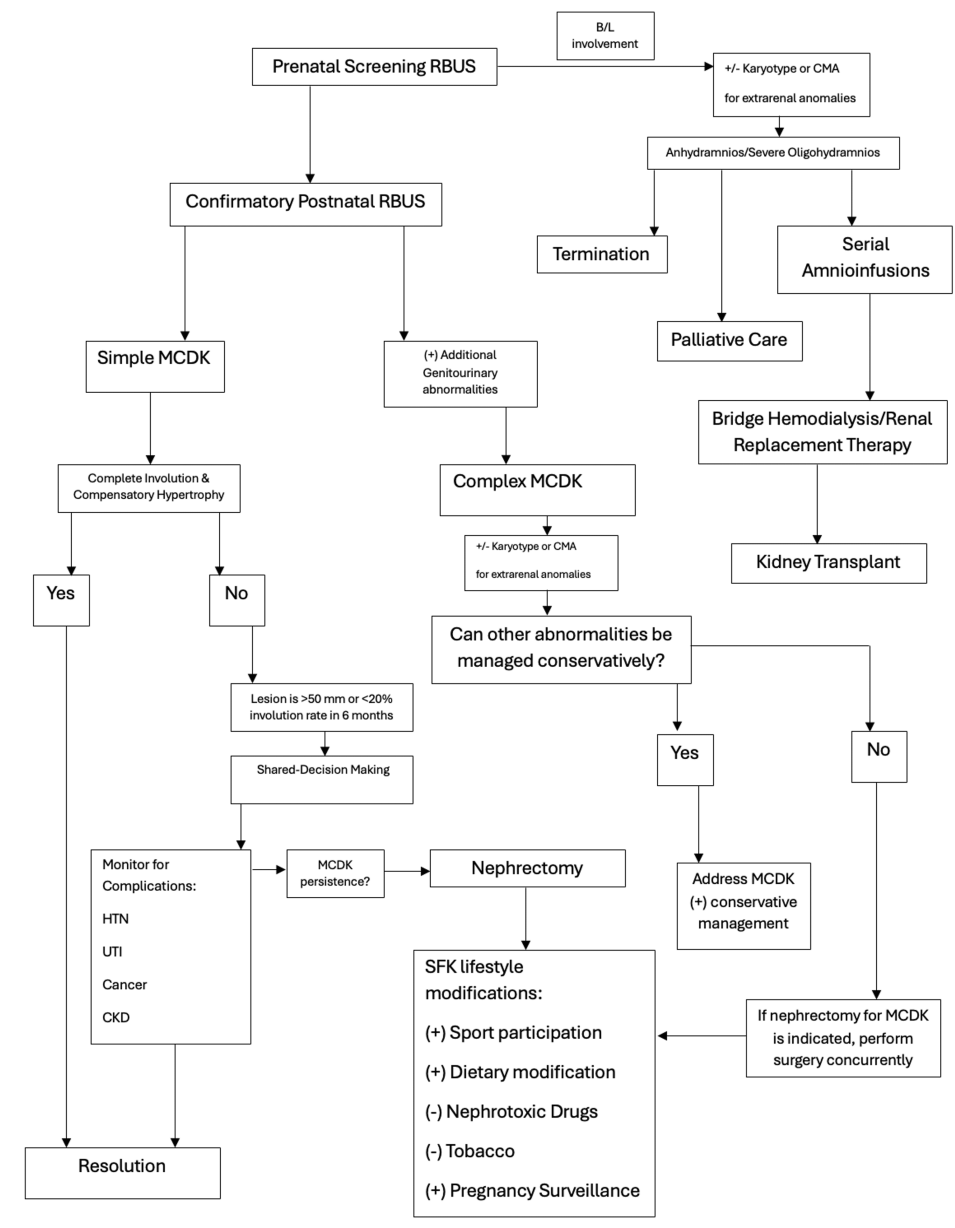
Summary of current guidelines in the management of MCDK RBUS: Renal Bladder Ultrasound; B/L: Bilateral; CMA: Chromosomal Microarray; MCDK: Multicystic Dysplastic Kidney; HTN: Hypertension; UTI: Urinary Tract Infection; CKD: Chronic Kidney Disease; SFK: Single Functioning Kidney

Minimally invasive surgical techniques for MCDK nephrectomy are preferred due to low complication rates nearing 2.7%. Laparoscopic nephrectomy is reliable, safe, and efficient, with reduced morbidity [23,29]. Figure 1 outlines the diagnostic, monitoring, and management approaches to varying presentations of MCDK.

Bilateral MCDK: Novel Management and Beyond

FRF, also known as the Potter sequence, is caused by bilateral renal agenesis, bilateral cystic diseases such as MCDK, or severe lower urinary tract obstruction [4,30]. Multiple isolated case reports and heterogeneous studies postulated that serial amnioinfusions, followed by dialysis and ultimately a kidney transplant once the infant reaches an appropriate weight of 8-10 kg, can manage anhydramnios. 

In response, the Renal Anhydramnios Fetal Therapy (RAFT) trial was instituted to determine the efficacy of amnioinfusions, safety, and short and long-term outcomes [11]. Studies describing management and outcomes of bilateral kidney anomaly and the RAFT trial are summarized in Table 1.

**Table 1:** Literature overview of FRF management in utero and beyond BRA: Bilateral Renal Agenesis; AI: Amnioinfusion; PD: Peritoneal Dialysis; VACTERL: Vertebral, Anal, and Cardiac abnormalities, Tracheoesophageal fistula, and Renal and Limb abnormalities; HD: Hemodialysis; BOO: Bladder Outlet Obstruction; CVVH: Continuous Veno-Venous Hemofiltration; FRF: Fetal Renal Failure

Reference	Study Design	Number of Maternal and Fetal Pairs	Fetal Diagnosis	Intrauterine Interventions	Postpartum Interventions	Results	Complications
Bienstock et al., 2014 [12]	Case report	1	BRA and anhydramnios	Five weekly AIs	PD at 36 hours of life, discharged at 19 weeks with daily PD	Nine months of age on PD, meeting all milestones, awaiting renal transplant (at time of publication)	Peritonitis at 22 weeks of age
Miyahara et al., 2016 [31]	Case report	1	Left renal agenesis and right dysplastic kidney, VACTERL	None	Continuous hemodiafiltration started at four days of age. Switched to PD at 75 days of age	Nine months of age on PD, awaiting renal transplant (at time of publication)	Laparoscopic omentectomy and four catheter unblocking surgeries
Haeri et al., 2017 [32]	Case series	2	Case 1: LUTO. Case 2: bilateral renal dysplasia	Case 1: 4 AIs Case 2: 5 AIs	Case 1: discharged home at two months on PD. Case 2: PD started on the second day of life	Case 1: Posterior urethral valves managed with ablation. Case 2: discharged home, 87th day of life on PD	Case 1: none. Case 2: necrotizing enterocolitis on day 30
Polzin et al., 2016 [33]	Case series	8	Cases 1-6: LUTO. Case 7: b/l renal anomaly, horseshoe, dysplastic. Case 8: b/l cystic dysplasia	Average of 14.3 AIs	Cases 3, 5, 6, and 7 died immediately or days postpartum	Cases 1, 2, and 4 survived on PD and received kidney transplants (all three currently alive at four years old)	Case 8: peritonitis/sepsis on PD and death day 94
Sheldon et al., 2019 [34]	Case series	2	Cases 1 & 2: BRA	Case 1: 4 AIs Case 2: 9 AIs	Case 1: started on PD at one day of life, discharged home on PD at four months old. Case 2: started on PD at 0 days of life, discharged home on PD at 3.5 months old.	Case 1: Received kidney transplant at 2.5 years old, on immunosuppression, excellent renal function at 5.5 years old with appropriate milestones. Case 2: Received kidney transplant at 20 months old, on immunosuppression, excellent renal function at 44 months old with appropriate milestones.	Case 1: none. Case 2: none
Riddle et al., 2020 [35]	Case series	8	Cases 1-8: BRA	Average of 11.4 infusions per case	3/8 cases that survived >48 hours received PD (2/8 also received hemodialysis)	Comfort care due to extreme prematurity 1/8 (no resuscitation), early neonatal demise (<48 hours) in 4/7, death before discharge for remaining 3/7 at 11, 33, and 187 days.	3/8 cases that survived >48 hours; died due to dialysis-associated complications
Miller et al., 2023 [11]	Prospective, nonrandomized clinical trial	18	Cases 1-18: BRA	Average of 11 infusions per case	Combination or isolated use of aquaphoresis, hemodialysis, and/or PD	1/18 prenatal demise, 17/18 live births, 14/18 cases received dialysis and survived >14 days, 4/18 cases long-term survivors (at the time of publication)	4/18 cases (long-term survivors); Development of grade 1 germinal matrix hemorrhage, two strokes, two seizures, and three cases of hypotension were seen
Magee et al., 2025 [30]	Case series	30	14/30 cases of MCDK, 6/30 cases of BOO, 5/30 cases of BRA, and 5/30 cases of unknown or a combination of conditions	Average of 9.5 AIs	PD for survivors beyond six months, 41% of survivors requiring the CVVH	Demise in 15 cases prior to six months postpartum, and 15 cases survived beyond six months. > six-month survivors required PD. 4/15 survivors received kidney transplants, 11/15 awaiting transplants or waiting to fulfill weight criteria for transplantation	41% of > six-month survivors required CVVH at some point of NICU stay due to leakage of dialysate or peritonitis (to clear infection)

Amnioinfusion may serve as a bridge to transplantation in select cases [30,33,34] but remains experimental. However, due to the 100% lethality of expectant management in FRF, this management option is viable despite being experimental [35]. The RAFT trial reports an uncertain long-term benefit, contrasting with smaller successful case series, raising ethical and psychosocial considerations.

Psychosocial impact and ethical considerations of MCDK

MCDK has a spectrum of presentations and managements; likewise, psychosocial impacts on the patient and their family depend on the extent of interventions. Psychosocial impact is driven by the emotional and economic burdens.

Financial Burden of Conservative Management

Conservative management of MCDK relies on continual monitoring. It is reported that RBUS follow-up costs $104 Australian dollars and $315 US dollars (USD), respectively [23,36]. Different centers have different protocols for determining follow-up intervals. At the Hospital for Sick Children in Toronto, Canada, patients are seen at 0 to 1 month postnatally, and then subsequently at three, six, and 12 months, followed by an annual or biannual imaging until 18 years of age (21 ultrasounds total). This center sees approximately 400 children with MCDK and congenital solitary kidney annually. The center instituted a new protocol and reduced the number of ultrasounds by 7, saving $46,200 USD/year [36]. These figures do not account for indirect costs borne by families, including transportation, parking, meals, missed school days for patients, and lost workdays for caregivers. 

Some authors state that a nephrectomy after a short surveillance period is better financially. Yamataka and colleagues compared the estimated total costs (ETC) of open nephrectomy, laparoscopic nephrectomy, and a group of patients with unilateral MCDK choosing observation. Participants in the observation group that did not experience involution in five years had the highest ETC [37].

Procedure-Associated Psychological and Iatrogenic Risks in Complex MCDK

Complex MCDK may require a diagnostic VCUG, which raises concerns due to radiation exposure, heightened cancer risk, and significant distress in children [38,39]. In toilet-trained children, VCUG is associated with significant pain and embarrassment compared to VUR surgery. Sedation is not always required for VCUG; however, it may be required for urethral catheter insertion (e.g., in cases of UTIs) [38]. Midazolam is routinely used, but can be associated with side effects including nausea, vomiting, dysphoria, or rarely respiratory depression, increasing procedural costs for families [40].

Psychological Consequences of Associated Chronic Complications and Cancer Survivorship

Poorly controlled hypertension may cause neurocognitive deficits and learning disabilities due to impaired cerebrovascular reactivity [41,42]. Adherence is often challenging, especially in adolescents; thus, caregiver support and a strong therapeutic alliance are essential [42,43]. 

Malignancy diagnosis and survivorship are associated with substantial psychosocial morbidity amongst affected children and their caregivers. Yardeni et al. report that post-traumatic stress disorder is prevalent in 8.7% of patients and 18.3% of their parents [44]. Depression is reported in 22% of pediatric cancer survivors. 7.6% of caregivers report depression, and 22% report anxiety [44]. In a study authored by Foster et al., adolescent WT survivors and their matched siblings were compared in psychological, educational, and social domains. Survivors were more likely to use special education services (25.5%), use psychoactive substances, and show increased difficulty in forming peer relationships. Socialization difficulties arise from frequent healthcare visits during early childhood, a critical period for social development [45].

Long-Term Psychosocial Impact of a Single Functioning Kidney

The Society for Maternal-Fetal Medicine (SMFM) focused on multiple aspects of life in children with single functioning kidneys (SFK) using data from the KIMONO study [46,47]. The authors concluded that an SFK in children is not a benign condition, such that by the age of 10, one in three enrolled children with either a congenital (primary) or acquired (secondary) SFK (i.e., nephrectomy) presented with indicators of renal injury, such as HTN, albuminuria, or took renoprotective medication. 

Renoprotective strategies require lifelong adherence. One of them is exercise, which carries a small risk of renal injury but reduces glomerular hyperfiltration. Dietary modifications, avoidance of obesity, nonsteroidal anti-inflammatory drugs, and tobacco also reduce hypertension risk. Life-long BP monitoring via ambulatory BP and urinalysis for microalbuminuria every five years is suggested [46,48]. Post-renal donor patients when pregnant carry a fivefold increased risk of gestational hypertension, diabetes, and preeclampsia [48]. Lifelong management imposes significant psychological and financial strain, requiring ongoing education and counseling.

Ethics & Shared Decision Making

FRF places a significant psychosocial burden on families, requiring transparent, patient-centered counseling to support informed decision-making. Non-directive counseling involving a multidisciplinary team consisting of fetal medicine obstetricians, nephrologists, pediatric surgeons/urologists, neonatologists, geneticists, psychologists, and social workers should be considered [18,30,33,49]. Ethical oversight from an Ethics Committee is important to maintain equity. Magee et al. suggest providing comprehensive medical and psychosocial care at one center [30].

A thorough prognostic timeline must be communicated with the emphasis on significant mortality and morbidity, inpatient stays in the neonatal intensive care unit (NICU) for approximately 60 days or more, surgical interventions, and the risks of renal replacement therapy. These interventions are offered in a small number of specialized centers, raising concerns of equitable access [50]. Equitable access may also be complicated by limited insurance coverage for NICU stays. In the US, a survey in 2005 estimated that the daily cost of NICU stay is $1250-2000 USD. On average, each preterm birth costs $51,600 USD [51]. The family must be counseled that the procedure may extend life for a lethal condition; however, it can result in prolonged suffering, as there is no clear evidence of benefit [50,52]. Isolated data from case reports has mixed implications. According to the RAFT trial, four infants demonstrated long-term survival but with prominent neurological deficits, i.e., strokes, seizures, and germinal matrix hemorrhages. Currently, these patients are awaiting criteria requirements for transplantation [11]. Polzin et al. and Magee et al. report that a total of seven patients survived kidney transplantation without complications [30,33]. Polzin et al. report that their surviving patients are all four years old and have an appropriate quality of life, besides requiring urethral catheterization and/or nutritional supplementation [33]. Publication bias may underreport unsuccessful cases [50]. Where appropriate, offer non-directive counseling and postnatal palliative care [18].

Limitations, recommendations, and future directions

MCDK is a well-studied entity with reviews that focus on various aspects of the disease. Our review is not a systematic review or a meta-analysis; however, it does focus on a novel aspect of MCDK, i.e., psychosocial and economic factors impacting the patients and their families. To our knowledge, other reviews do not address these concerns on a comprehensive level. This review urges providers to consider an individualized approach by educating the patients and their families on the importance of follow-up and renoprotective strategies, whether a conservative or a surgical approach is chosen. Our review does not systematically assess the long-term outcomes of the novel fetal therapies due to limited data on the outcomes of these therapies. As the body of knowledge continues to expand, a better understanding of the efficacy of these interventions will emerge. Our hope is that this review will benefit future researchers in their scientific endeavors by providing a summary of such interventions.

## Conclusions

MCDK represents a congenital renal anomaly comprising a broad spectrum of clinical presentations ranging from unilateral cases that undergo spontaneous involution to complex cases resulting in perinatal morbidity and mortality. Advancements in pediatric healthcare have improved diagnosis, management, and risk stratification methods, resulting in an individualized approach. While conservative management and surveillance remain the standard of care for unilateral cases, if contralateral anomalies are found, a combined surgical intervention may be required. In expectant management of bilateral MCDK, perinatal mortality is assured; however, emerging experimental interventions are now being explored to improve prognosis. Given potential complications and long-term monitoring requirements, the associated diagnostic and therapeutic interventions impose a considerable psychosocial, ethical, and economic burden on affected patients and their families. This warrants a personalized multidisciplinary approach ensuring shared decision-making and patient-centered management care plans.
